# Toxicity Assessment of *Asteraceae Centaurea Repens* L Extract in Mice

**Published:** 2017

**Authors:** Mohammad Moradi, Faraz Mojab, Sepideh Arbabi Bidgoli

**Affiliations:** a *Pharmaceutical Sciences Research Center, Islamic Azad University (IAUPS), Pharmaceutical Sciences Branch, Tehran, Iran. *; b *Department of Pharmacognosy, School of Pharmacy, Shahid Beheshti University of Medical Sciences and Health Services, Tehran, Iran. *; c *Department of Toxicology & Pharmacology, Islamic Azad University, Pharmaceutical Sciences Branch (IAUPS), Tehran, Iran.*

**Keywords:** Asteraceae Centaurea repens L, Acroptilon repens, Rhaponticum repens, Russian knapweed, Liver toxicity, Asteraceae

## Abstract

The species Asteraceae Centaurea repens (Asteraceae), known as Acroptilon repens, and Talkhe in persian is used in folk medicine as an emetic, anti-epileptic, and anti-malaria herb in many parts of the world but its toxic effects have not determined yet. This study aimed to evaluate the acute and subchronic toxicity of this extract to find its possible adverse health effects through clinical, hematological, biochemical, and histopathological endpoints in both gender of mice. Aerial parts of the plant were air-dried and the terpene extract of aerial parts of plant was provided by percolation using methanol, petroleum ether, and diethyl ether. All clinical, biochemical and histopathological changes were assessed in appropriate endpoints and compared with control group. Although no mortality was seen in acute study by administrating doses up to 2000 mg/kg, repeated dose study on 1000 mg/kg doses in 28 days in both genders showed liver necrosis and rise of liver enzymes (p-value < 0.05). Histopathological studies didn’t show any other organ toxicity in dosed up to 1000 mg/kg. At the same time this study showed for the first the antihyperlipidemic properties of the aerial extract of Acroptilin in mice model. The pharmacological and histopathological results of the present study proved that the total parts of *Acroptilon repens* could be studied for supporting the traditional assertion in folk medicine to heal hyperlipidemia, diabetes, and cancer in lower doses although we performed the present study and concluded liver toxicity by subchronic use of *Acropitolon repens* extract.

## Introduction

Acroptilon repens (Asteraceae) is one of the most invasive and ecologically threatening weed species with considerable allelopathic effects (1)Allelopathy is a well known negative effect of some chemicals which could be produced by one plant on neighboring plants, frequently mediated through root exudates and other plant leachates (2) *Acroptilon repens* (Rhaponticum repens), with the common name of Russian knapweed with intense and stiff root invading adjacent plants (3) is resistant to difficult climate conditions and grows in many countries worldwide including Iran, western Turkestan, Mongolia, Turkish Armenia, Asia Minor , United States and Canada (4).

**Table 1:** Organ weights in high dose treatment and controls after 28 days of study

**Organs**	**gender**	**Treatment** **N: 3**	**Control** **N: 3**	**p-value**
		**Mean of organ weights (SD) ,[g]**		
Kidney	F	0.243 (0.006)	0.243 (0.006)	1.000
	M	0.247 (0.006)	0.243 (0.006)	0.519
liver	F	1.243 (0.07)	1.070 (0.026)	0.017*
	M	1.283 (0.035)	1.043 (0.040)	0.001**
lung	F	0.1733 (0.006)	0.1733 (0.006)	1.000
	M	0.193 (0.021)	0.170 (0.010)	0.155
spleen	F	0.110 (0.100)	0.113 (0.006)	0.643
	M	0.107 (0.006)	0.103 (0.006)	0.519
heart	F	0.193 (0.021)	0.187 (0.006)	0.621
	M	0.190 (0.010)	0.183 (0.116)	0.492

**Table 2:** Changes in biochemical factors in high dose group after 28 days of study

**Biochemical Variables**	**sex**	**Treatment** **N: 3**	**Control** **N: 3**	**p-value**
		**Mean (SD)**		
ALP U/l	F	303.000 (115.326)	21.667 (3.519)	0.013*
	M	393.000 (37.510)	22.667 (6.506)	<0.001***
ALT U/l	F	201.334 (210.719)	167.334 (33.081)	0.796
	M	121.000 (57.158)	148.667 (36.019)	0.517
AST U/l	F	461.334 (369.673)	56.000 (3.606)	0.130
	M	472.334 (90.456)	40.334 (7.095)	0.001**
HDL	F	47.667 (6.351)	10.000 (2.000)	0.001**
	M	71.000 (8.718)	15.334 (4.163)	0.001**
LDL	F	8.000 (2.646)	119.334 (8.145)	<0.001***
	M	29.334 (8.621)	120.667 (21.548)	0.002**
TG	F	89.667 (7.234)	54.000 (6.245)	0.003**
	M	57.334 (13.317)	9.540 (9.539)	0.428
FBS (mM)	F	79.000 (13.747)	115.333 (3.512)	0.011*
	M	116.000 (26.907)	119.000 (2.646)	0.857

**Figure 1:**
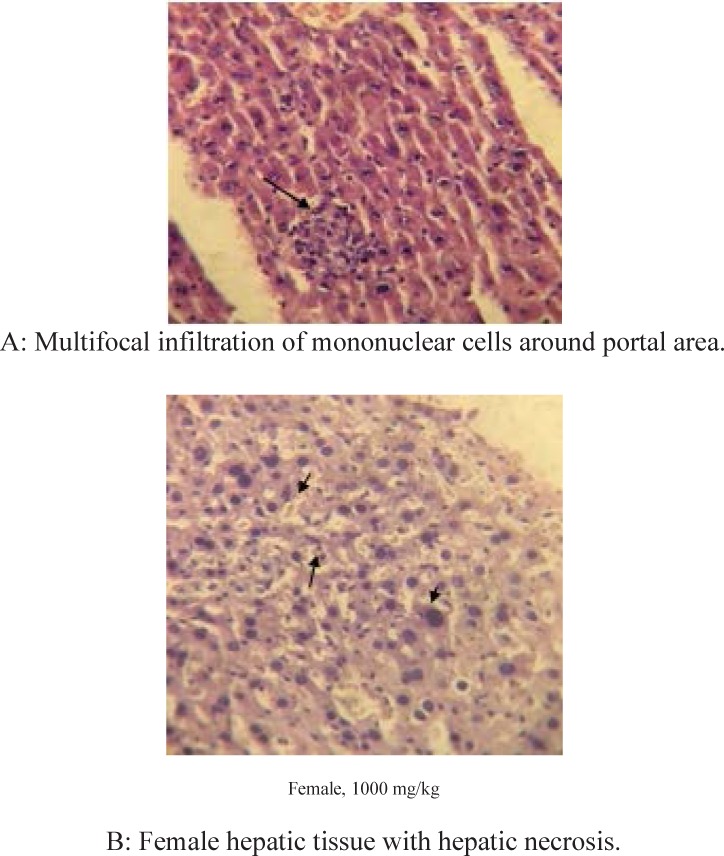
Hepatotoxic effects of Asteraceae Centaurea repens L extract in high dose (1000 mg/kg) female mice after 28 days of study (x40).

Chronic ingestions of this weed by horses in different regions have been reported to create equine nigropallidal encephalomalacia (ENE), which is associated with a movement disorder Parkinson disease (PD) signs (5). The disorder is characterized by a fixed wooden facial expression with hypertonia of the muscles of the muzzle and face, idle chewing, tongue flicking, and impaired eating and drinking, followed by hypokinesia and death (6).

Repin, an essential ingredient extracted from Russian knapweed, is a kind of sesquiterpene lactons which has the most concentration and toxicity among several sesquiterpene lactons existing in this plant extract from other parts of the world (7). Repin structure contains an a-methylenebutyrolactone moiety (5) and epoxides, considered as a highly reactive electrophile that can easily conjugate with various biological nucleophiles, such as proteins, glutathione, and DNA (5), (8). Ultimately it leads to striatal extracellular dopamine denervation, oxidative stress, and degeneration of nigrostriatal pathways. Neuropathological studies revealed bilateral necrosis of the anterior globus pallidus and the substantia nigra (9). Sesquiterpenoids have been suggested to cause intense neurotoxic effects on primary cultures of fetal rat brain cells (10) without any *in-vivo* findings.

Despite the above facts regarding the possible toxic potentials of Repin, an essential ingredient extracted from Russian knapweed, its root has been used for many years as emetic, anti-epileptic and anti-malaria remedy in Uzbekistan, Kazakhstan and Azerbaijan’s traditional medicine (11) and there is no study on pharmacologic and toxicological properties of its aerial parts therefore we motivated to evaluate the target organ toxicity of *Acropitolon repens* terpene aerial extract in mice for the first time. To evaluate the acute and repeated dose oral toxicity, we used OECD 425 and 407 guidelines respectively for regulatory decisions and possible commercialization.

## Experimental

*Asteraceae Centaurea repens L extract*

The plant material was collected from Markazi province, Nobaran region in June 2015. The voucher specimen of the plant is available at the Herbarium of Islamic Azad University Pharmaceutical Sciences Branch (IAUPS), Tehran, Iran.

Aerial parts of the plant were air-dried in 10 days and the total weight of plant was reduced from 900 g to 700 g. The terpene extract was provided by method of percolation using methanol, petroleum ether, and diethyl ether (1:1:1) during 6 days. After filtration of extract, the organic solvents were excluded by rotary evaporator in 45 ºC, and a sticky powder was provided because of existing wax in it. So, this time, just methanol was added to powder and the provided solution was kept in -15 ºC refrigerator for 48 h. Finally the waxes accumulated on the top of solution were removed; a fine powder was obtained and used for toxicological tests after utilizing rotary evaporator for the present study.

*Experimental animals and housing conditions*

Experimental healthy mice in both genders were obtained from Pasture Institute of Iran at 6-8 weeks of age and 25.1 ± 1.9 g body weight. Female and male mice were individually housed in separate quarters in solid bottom cages. Individual animals were identified by color coding, the animal number and group number also appeared on the outside of each cage to preclude mix-up. The animal room environment was controlled (targeted ranges: temperature 22-25 °C, relative humidity 30-70%) and monitored daily. The photo-cycle was 12 h light and 12 h dark. Upon arrival all animals were submitted to a general physical examination and all were found healthy and were admitted. The animals were conserved on a 12 h dark/light cycle at about 22 ± 3 °C and assigned free access to standard laboratory diet (Pars Co.) and tap water during the experiments. Diet and water were offered ad libitum throughout the acclimatization and study periods. The cage cleaning schedule, air filtration and recirculation, health checks and facility maintenance were carried out in accordance with the IAUPS standard operating procedures, and such activities were recorded in the animal room records. Animals were housed and maintained according to the Ministry of Health of Iran for the care and use of laboratory animals, CCAC guidelines for care and use of experimental animals and IAUPS standard operating procedures. Investigation using experimental animals contained a statement confirming the adherence of the research to the principles of laboratory animal care (NIH publication, revised in 1985) and approved by the ethical committee of IAUPS.

*Animal Selection/Randomization*

The test population of animals was selected from newly arrived, previously unused mice. The method of randomization was based upon the random selection of numbers generated from a set of numbers without replacement.

*Preparation of Animals*

All animals used for the limit test were fasted over-night. Food but not water was withheld from 5: 00pm. On the day preceding dosing. In order to minimize stress caused by fasting, the animals were offered a 10% w/v aqueous solution of glucose during this period.

## Experimental Procedures

*Acute test*

To determine the toxic potential of the test article by oral ingestion, acute test was considered according to the OECD 425 guideline. This study permitted an estimation of the LD50 of the test article and the results were allowed for ranking and classification of the test article according to the globally harmonized system of classification and labeling of chemicals (GHS), and OPPTS toxicity categories guidelines.

*1. Limit Test*

The chemical composition indicated that the test article was likely to be non-toxic; therefore, a decision was made to proceed with the Limit test. First animals (1 mice and 1 rat in both genders) were dosed at 5000 mg/kg. Since these animals survived, four additional animals in each strain and gender were sequentially dosed at approximately 48 to 72 h intervals. A total of 20 animals were tested.

*2. Test Article Preparation*

Since the test article was a granule, they were used on “1 g/mL” suspension. The vehicle for preparing this suspension was distilled water. 

*3. Dose Administration*

The animals were dosed at 2000 mg/kg (0.2 mL/kg by volume). The individual doses of the test article were individually calculated for each animal based on the body weight of the animals. All doses were administered orally using a feeding cannula, inserted into the stomach of the animals.

*4. Observations during In-Life Phase*

The animals were individually observed once during the first 30 min after dosing and periodically during the first 48 h following dosing (with special attention given during the first 4 h). Observations once daily were carried out for the rest of study. The animals were observed for 14 days after the dosing. Cageside observations were directed towards any changes in the skin and fur, eyes and mucous membranes, and also respiratory, circulatory, autonomic and central nervous system, and somatomotor activity and behavior pattern. Particular attention was directed to any observation of tremors, convulsions, salivation, diarrhea, lethargy, sleep and/or coma. Any symptoms of toxicity and deaths were recorded daily for the entire study period and the entries were monitored. The body weights of the animals were determined prior to test article administration (*i.e.* day 0), on day 7, day 13 and again on day 14. 

*Post Mortem Examination*

Gross necropsy was performed on each mouse at the end of the 14 day observation period and necropsy included an examination of: external surfaces of the body; all orifices; cranial cavity; external surfaces of the brain and spinal cord; nasal cavity and paranasal sinuses; thoracic, abdominal, and pelvic cavities and viscera.

*Subchronic toxicity study*

Three test groups and a control group were used, but from our first assessment acute oral toxicity study, no effects were observed at a dose of 2 mL/100 g and the test substance was categorized as practically non-toxic agent or category 5 in GHS classification. Except for treatment with the test substance, animals in the control group were handled in an identical manner to the test group subjects. As the vehicle used in administering the test substance was water, the control group received the same volume of water. Dose levels were selected according to the existing toxicity data available for the test compound or related materials from our first studies. 

The highest dose level was chosen with the aim of inducing toxic effects but not death or severe suffering. Thereafter, a descending sequence of dose levels was selected with a view to demonstrating any dosage related response and no-observed-adverse effects at the lowest dose level (NOAEL). Two to four fold intervals are frequently optimal for setting the descending dose levels and addition of a fourth test group is often preferable to using very large intervals (*e.g.* more than a factor of 10) between dosages. On the basis of this concept dose levels were determined as Low dose group (250 mg/kg), medium dose group (500 mg/kg) and high dose group (1000 mg/kg) from terepene extract on the basis of their body weights once daily for 6 days per week during a period of 28 days. Distilled water was administered to negative control group of animals. In the presence of observed general toxicity (*e.g.* reduced body weight, liver, heart, lung or kidney effects, *etc.*) or other changes that may not be toxic responses (*e.g.* reduced food intake, liver enlargement), observed effects on immune, neurological or endocrine sensitive endpoints was interpreted with caution in this study .

The observation period was 28 days. Animals in a satellite group scheduled for follow-up observations which were kept for at least 14 days without treatment to detect delayed occurrence, or persistence of, or recovery from toxic effects. 

*Clinical examinations*

Clinical signs were observed and weights were measured and recorded once daily in acute study and once weekly in subchronic study. The recording items were divided to three categories:

Cageside Observations contained home cage activity, feces color, feces amount, feces consistency, urine color, urine amount and behavior while removing from cage.

Neurological Examination analyzed tail elevation, abnormal gait, ataxic gait, and head position.

Physical Examination included death, hair coat, mucus membrane/eye/skin color, body temperature, respiratory rate, respiratory character, lacrimation, amount of salivation, and eye prominence.

*Biochemical assays*

Biochemical parameters were measured in three female and three male mice of high dose group and control group with an automated biochemical analyzer in the same laboratory and consisted of fasting blood glucose (FBS), low density lipoprotein (LDL), high density lipoprotein (HDL), fasting triglycerides (TG), aspartate aminotransferase (AST or SGOT), alanine aminotransferase (ALT or SGPT), and alkaline phosphatase (ALP in day 28 or last day of study).

*Histopathological studies*

Various organs from respiratory system (lung), cardiovascular system (heart), lymphatic system (spleen), urinary system (kidneys), digestive tract (liver) were removed from 3 female and 3 male mice of high dose group and control group whose serums were assayed for biochemical studies. Organ weights were recorded and absolute and relative organ weights were compared in each group with related control. The tissues were fixed in 10% buffered formalin and dehydrated in graded series of alcohol, cleared in xylene and embedded in paraffin wax. Multiple sections from each block were prepared at 5 µm and stained with haematoxylin and eosin (H&E).

*Statistical analysis*

Values were expressed as mean ± SD. Firstly, evaluation of homogeneity of variances was done to compare groups.

If variances were not significantly different, the data were analyzed by one-way analysis of variance (ANOVA) and the Student’s *t*-test. When variances were assumed significantly different, Man-Whitney U test for comparison of two variables and Kruskall-Wallis H test for comparison of more than two variables were exerted. A significant difference was accepted with *P *< 0.05. SPSS 21 was the software; all statistical methods were performed with.

## Results

*Acute oral toxicity*

One single oral dose of 2000 mg/kg was administered to all treated animals. No deaths and no signs of toxicity were observed in the first 48 h of administration. According to the lack of mortality in mice at the limit test, the LD50 value for terpene extract of *Acropitolon repens* was supposed to be higher than 2000 mg/kg of body weight and this extract was categorized as practically non-toxic agent. Observations were continued for the next 14 days of this study. Because all animals found healthy with normal physical activities during the next 14 days of study, doses of 250, 500, and 1000 mg/kg were considered as three dose levels for subchronic toxicity study.

*Subchronic toxicity study*

*Food and water consumption and weight changes*

Food consumption was not significantly different among control and treatment groups (*N/S*) also no remarkable changes in levels of water consumption were observed in all dose groups (*N/S*). Weights of animals from all three treated groups were recorded once weekly. No significant weight change was detected for the duration of the study (*N/S*).

*Survival and clinical signs*

All animals were survived during this study in cases and control groups. Out of different daily cage side observations no clinical sign of toxicity was recorded in dose groups and control.

*Body organ weight*

After subchronic study, increased size and weight of liver was distinctly distinguished in two genders in high dose treatment groups. As mentioned in Table 1, significant liver weight gain was observed (1.243 ± 0.07 g vs. 1.070 ± 0.026 g), (*P *= 0.017) and increased weight of liver in male high dose group was seen too (1.283 ± 0.035 g vs. 1.043 ± 0.040 g), (*P *= 0.001). Exact weight of organs after 28 days of study in female and male high dose groups has been demonstrated in Table 1.

*Hematological factors*

Blood samples of animals were analyzed at days 14 and 28 of study and compared at each endpoint with controls. No significant change was observed in each dose level. Data were not shown.

*Biochemical studies*

After a 28 day study, serum samples of animals were analyzed and compared with controls. Significant raise in ALP was seen in high dose groups of females and males (*p *= 0.013 and **< **0.001 respectively). Remarkable change of AST level was recorded just in males of high dose group (*p *= 0.001). Meaningful decline in FBS was gained only in females of high dose groups (*p *= 0.011). In the mean time and only in high dose group, lipid profile of animals underwent surge changes that means HDL strongly rose in females and males of high dose groups (*p *= 0.001 in both genders), LDL decreased dramatically in males and females (*p *= 0.002 and < 0.001 respectively) and TG didn’t change meaningfully in both genders. These changes showed significant metabolic effects of this extract in the 28 days repeated dose administration. Other biochemical changes were considered non-significant and not demonstrated in Table 2.

*Histopathological studies*

Histopathological studies which were performed on H&E stained slides at 28 day of study, didn’t show any abnormal changes in all organs except the liver of both genders in 1000 mg/kg dose group. Liver necrosis was detected in the liver of both genders after 28 days repeated dose administration of 1000 mg/kg. Figure 1a shows hepatocellular necrosis and multifocal infiltration of mononuclear cells around portal area. Figure 1b shows the same feature in female animal.

## Discussion

Acroptilon (Centaurea solstitialis) is a native plant in Iran, Turkey, central Asia, and China that can be a problematic weed in agricultural settings (12), (13). Although its traditional uses have been considered for many years as a folk remedy throughout many areas worldwide (11), its antidiabetic and antioxidant properties have been recently considered (14). Through isolation of new sesquiterpenoid alkaloid together with already known guanine type sesquiternoids from the aerial part of Acroptilon repens with potent cytotoxic activities against tumor P-388 cell line (15), C6 cells and HeLa cells (16), its possible cytotoxic potentials for future anticancer applications could be considered soon. As the toxicity profile of this plant in acute and subchronic tests have not been determined yet, this research aimed to provide some useful information about its toxic properties in acute and repeated dose models. In fact this research enabled us to judge better about the safety issues of *Acropitolon repens *in mice model through OECD guidelines using clinical, biochemical, and histopathological evidences as followings:

*Acute oral toxicity*

According to the acute model, lack of mortality and any other clinical and post mortem observations signs of toxicity in the first 24 h of administration in doses up to 2000 mg/kg, this plant could be categorized as category 5 materials according to the globally harmonized system of classification and labeling of chemicals criteria (17). The extract of this plant from Markazi province, Nobaran region showed no hazardous effects in both mice genders in acute doses.

*Subchronic Toxicity*

After categorization of the plant, we exerted subchronic method and collected information of toxic responses in comparison to control groups. Lack of neurotoxic effects according to our repeted oral dose study in all dose groups, suggests lower levels of repine in the extract of plants which grow in this region of Iran but this matter extract should be analyzed and compared with similar specied from other geographical regions in Iran and other countries in next studies. This observation is in accordance with Mettler *et al.* hypothesis who assumed that repin intoxication reduces striatal and hippocampal glutathione and inhibits the release of dopamine in horse without affecting its uptake in mice (18).

*Liver toxicity*

Our findings revealed, subchronic high dose administration of terpene extract of this plant in doses of 1000 mg/kg results in dramatic raise of some liver enzymes (ALP, AST) as nonspecific biomarkers of hepatotoxicity, significant increase in liver organ weights, hepatocellular necrosis, and multifocal infiltration of mononuclear cells around portal area in both genders. These serial signs warned us about its possible human risk to liver damages in high doses in repeated dose administrations. One recent study by Zhan ZJ *et al.* declared a novel susquiterpenoid alkaloid in *Acropitolon repens* extract with antitumor effects on tissue culture through hypoxic mechanism (15). The damages were created in liver tissue by terpene extract, likely depended to hypoxia mechanism or extensive ROS formation and oxidative stress by the extract or its possible metabolites (8). Due to hypoxic potentials of Acroptilon extract (15), it could be assumed that repeated dose oral administration of plant extract caused hypoxia in hepatocytes of oxidative stress which leaded to hepatocellular distraction and focal necrosis. Histopathological studies on other organs didn’t show any abnormal change in all organs of both genders in doses up to 1000 mg/kg except the liver in high dose group explained above. 

*Pharmacological Effects*

Most of the herbal derived compounds have long been known for their potential pharmaceutical effects but very limited pharmacological researches have been done on pharmacologic effects of *Acropitolon repens*. This study showed the lipid and glucose lowering effects of this plant material for the first time in both genders of mice. HDL levels increased in both genders around 5 times in comparison to control group (*P* = 0.001) and around 10 times decrease in LDL levels (*P* ≤ 0.001) was seen in high dose group surprisingly. These preliminary observations should be regarded as a new hypothesis on its possible metabolic effects in high fat diet animal models.

On the other hand, mild FBS lowering effects of this extract in female mice ( *P* = 0.011) which is in accordance to a recent study (14) suggest its possible synergistic effects in combination of other medicinal herbs of Asteraceae which should be carefully tested in a new setting in diabetic animal model. 

## Conclusion

Since there is no controlled study on Acroptilon repens total extract toxic effect, we conclude the safety and lack of toxicity of this extract for short term uses up to 2000 mg/kg and in repeated doses up to 1000 mg/kg except liver toxicity in mentioned dose. Hence, it is necessary to establish the scientific basis for the therapeutic actions of this folk medicine as it may serve as the source for the development of more effective drugs for hyperlipidemia, diabetes, and cancer. Relative safety of this plant material may play an important role in the treatment of several diseases from hyperlipidemia to various types of cancers because many present clinically effective pharmaceuticals are developed from plant-derived ancestors in the history of medicine. The pharmacological and histopathological results of the present study proved that the total parts of Acroptilon repens could be checked up to 500 mg/kg dosed (no observed adverse effect level/NOAEL) for future studies and supporting the traditional assertion of this regional herb (Talkhe) in central Iranian folk medicine and many other parts of the world. 
